# Psychometric evaluation of the traditional Chinese version of PedsQL^™^ 3.0 cardiac module scale in adolescents with congenital heart disease: reliability, validity, measurement invariance, and adolescent-parent agreement

**DOI:** 10.1186/s12955-023-02121-1

**Published:** 2023-05-05

**Authors:** Yong-Chen Huang, Yueh-Chih Chen, Bih-Shya Gau, Jou-Kou Wang, Shu-Hui Chang, Hsiao-Ling Yang

**Affiliations:** 1 Institute of Epidemiology and Preventive Medicine, National Taiwan University, No. 17, Xu-Zhou Road, Taipei City, 100 Taiwan; 2 School of Nursing, College of Medicine, National Taiwan University, No. 1 Jen-Ai Road, Section 1, Taipei City, 100 Taiwan; 3 Department of Nursing, National Taiwan University Hospital, No. 7, Chung-Shan South Road, Taipei City, 100 Taiwan; 4 Department of Pediatrics, National Taiwan University Hospital, No. 7, Chung-Shan South Road, Taipei City, 100 Taiwan

**Keywords:** Congenital Heart Disease, Confirmatory Factor Analysis, Health-related Quality of Life, Adolescent

## Abstract

**Background:**

In recent decades, 95% of children with congenital heart disease (CHD) can survive to adolescence and adulthood. However, adolescents with CHD are prone to poorer health-related quality of life (HRQoL). It is imperative to develop a reliable and valid instrument for health professionals to monitor the HRQoL. This study aims to: (1) evaluate the psychometric properties of the traditional Chinese version of Pediatric Quality of Life™ 3.0 Cardiac Module (PedsQL-CM) and measurement invariance across adolescents with CHD and their parents; and (2) investigate the adolescent-parent agreement in HRQoL.

**Methods:**

A total of 162 adolescents and 162 parents were recruited. Internal consistency was examined using Cronbach’s alpha and McDonald’s Omega. The criterion-related validity was evaluated with intercorrelations between the PedsQL-CM and PedsQL™ 4.0 Generic Core (PedsQL-GC) Scale. The construct validity was examined by second-order confirmatory factor analysis (CFA). Measurement invariance was evaluated using the multi-group CFA. The adolescent-parent agreement was analyzed with the intraclass correlation (ICC), paired t-tests, and Bland–Altman plots.

**Results:**

PedsQL-CM showed acceptable internal consistency (self-reports 0.88, proxy-reports 0.91). The intercorrelations were medium to large effect size (self-reports 0.34–0.77, proxy-reports 0.46–0.68). The CFA supported the construct validity (CFI = 0.967, TLI = 0.963, RMSEA = 0.036, 90% CI = 0.026–0.046, SRMR = 0.065). The multi-group CFA proved scalar invariance between self and parent proxy-reports. Parents significantly underestimated their adolescents’ HRQoL in cognitive problems (Cohen’s *d* = 0.21) and communication (Cohen’s *d* = 0.23) subscales, while there was a negligible difference in total HRQoL (Cohen’s *d* = 0.16). ICCs were poor to moderate effect size with the highest and lowest agreement in heart problems and treatment subscale (ICC = 0.70) and communication subscale (ICC = 0.27), respectively. The Bland–Altman plots showed lesser variability in the heart problem and treatment subscale and the total scale.

**Conclusion:**

The traditional Chinese version of PedsQL-CM has acceptable psychometric properties to measure disease-specific HRQoL in adolescents with CHD. Parents may be proxies for adolescents with CHD to rate total HRQoL. When the patient-reported score is the primary outcome, the proxy-reported score could serve as a secondary outcome for research and clinical evaluation.

## Introduction

With substantial medical progress, 95% of children with congenital heart disease (CHD) can survive to adolescence and adulthood in recent decades [1]. Despite receiving successful surgical treatment, these patients need lifelong follow-up since they still suffer from physical limitations. Postoperative cardiac residuals and sequelae, including residual shunt, stenosis, obstruction, arrhythmias, ventricular dysfunction, and hemodynamic abnormalities, etc., may affect psychosocial functions [2–4]. Comorbidities and worsened cardiac function are associated with mental illness and lower health-related quality of life (HRQoL) [3, 5]. Previous studies showed that patients with comorbidities had significantly worse HRQoL compared with those without comorbidities [6, 7]. Furthermore, patients with chronic comorbidities, such as coronary artery disease and lung disease, had demonstrated as a mediator between CHD and depression [5]. Adolescents with CHD are prone to poorer HRQoL in that they are learning to take responsibility for self-care and might struggle with difficulties such as uncertainty about the future, how to build self-esteem, and use coping strategies [8, 9]. Although a high score of HRQoL can indicate successful management of disease burden for these patients [10], it was found that the HRQoL significantly decreased for adolescents and adults with CHD in a short three-year follow-up [11]. Furthermore, a recent study across 15 countries found that these patients had lower quality of life in Asian countries [12]. Therefore, it is imperative to develop reliable and valid instruments, especially the Asian language versions, for health professionals to monitor the needs of adolescent CHD patients with regular evaluations by HRQoL.

HRQoL is a multidimensional construct that reflects “how well a person functions in their life and his or her perceived wellbeing in physical, mental, and social domains of health” [13]. HRQoL can be measured by generic and disease-specific scales. The former summarizes an overall health condition in healthy and patient populations, while the latter more sensitively reflects the change realized by interventions in the specific disease population [14]. A recent systematic review and meta-analysis showed that five generic HRQoL scales had been applied to the CHD surgical population, while only Pediatric Quality of Life Inventory™ 3.0 Cardiac Module (PedsQL-CM) has been used for cardiac-related HRQoL [15]. This scale assesses the impact of symptoms, perceived physical appearance, treatment anxiety, cognitive problems, communication, and treatment adherence on HRQoL in patients two to 18 years of age, and it only requires 10–15 min to complete both PedsQL-CM and Pediatric Quality of Life Inventory™ 4.0 Generic scale (PedsQL-GC) [16]. PedsQL-CM has been translated into many languages for ease of usage [17–21], demonstrating its generalizability for different populations of pediatric patients with cardiac defects. However, despite the widespread development of PedsQL-CM in European and American countries, studies focusing on the Asian population are limited. The psychometric properties of the traditional Chinese version of PedsQL-CM have yet to be investigated.

The PedsQL-CM can be done by self-reports and parent proxy-reports. A parent proxy-report may be especially useful when the adolescent is unwilling or unable to conduct the questionnaire [22]. Gathering information by HRQoL from both adolescents and their parents can help healthcare providers understand the impacts of CHD on adolescents’ HRQoL and parental thoughts [16, 23, 24]. While patients’ self-reported data best present their actual HRQoL, adolescents who grow up with congenital or chronic diseases may not have experienced better health and overestimate their HRQoL. On the other hand, parents may offer a broader perspective of their children’s HRQoL based on social referencing [25, 26]. Additionally, when parents and adolescents have significantly different perceptions of HRQoL, such as parents underestimate adolescents’ physical function and adopt an overprotective parenting approach, medical professionals can help adolescents and parents clarify their feelings and encourage them to understand each other’s viewpoints, which may consequently improve parents’ parenting style and then improve the HRQoL for adolescents with CHD [27, 28].

Studies have shown that adolescents in various pediatric populations with chronic illness and their parents may rate the questionnaires differently [17, 29, 30]. The discrepancy may be associated with observable/non-observable symptoms that parents tend to underestimate the psychosocial functioning and overestimate the physical functioning [31, 32]. One possible reason for the disagreement concerns the related factors that may influence the ratings, such as age, gender, and health status [15, 33], and another may result from measurement non-invariance. Ideally, a parent proxy-report asks the parents to answer as they think their children would. Nevertheless, it has been suspected that the parents may mistakenly respond to their perceptions of their children as the standard parent reports, making the ratings between parents and adolescents noncomparable [33]. The actual group difference between self and parent proxy-reports can be inferred only when measurement invariance ensures that the two questionnaires measure the same construct with the same factorial structure [34, 35]. However, to our knowledge, little literature checked the factorial structure and measurement invariance of PedsQL-CM, making the findings of agreement or disagreement uncertain.

This study aimed to: (1) translate and evaluate the psychometric properties of the traditional Chinese version of PedsQL-CM and measurement invariance across adolescents with CHD and their parents; and (2) investigate the adolescent-parent agreement in HRQoL.

## Methods

### Participants and procedures

A cross-sectional study was performed and participants were recruited during an outpatient clinic visit at the National Taiwan University Hospital. Both adolescents and parents were invited to fill out the questionnaires. Adolescents who were (1) aged between 12–18 years, (2) diagnosed with CHD in infancy, (3) free from other congenital abnormalities and mental retardation, and (4) Chinese speakers, were included in the study. Those who received cardiac surgery or catheterization within the past three months and those whose parents were unable to participate in this study were excluded. Informed consent was obtained from both adolescents and their parents after introducing the study purpose and procedure, and ensuring anonymity.

We screened adolescents according to their age and disease diagnosis from the medical record, then understood their current disease progression and if the accompanying person was their parents during their clinic visit. After confirmed the adolescents and their parents met the inclusion criteria, the researcher introduced the research objectives and methods to the patients and their accompanying parents, invited them to participate in this study, and obtained informed consent from both adolescents and their parents. Adolescents and their parents completed the questionnaire separately and independently in a clinic room after the outpatient visit. For those who could not complete the questionnaire after the outpatient visit, they brought the questionnaire back home to fill out and mailed it to researchers using the self-addressed stamped envelope. In this case, we reminded them to fill out the questionnaire independently to offer valuable data and contacted them by telephone if they didn't return the questionnaire within two weeks.

### Measures

#### PedsQL™ 3.0 cardiac module (PedsQL-CM) scale

We used the 13–18 years version of the PedsQL-CM in this study [16]. It contains 27 items with six factors: heart problems and treatment (7 items), perceived physical appearance (3 items), treatment anxiety (4 items), cognitive problems (5 items), communication (3 items), and drug-related treatment (5 items). A 5-point Likert scale was used, and the score of each item was transformed into a 0–100 scale. A higher average score indicates a better level of HRQoL. As only the adolescents who were currently taking medication needed to complete the drug-related treatment subscale, we focused on the other five subscales, which every participant had experienced in this study.

The translation process of the traditional Chinese version of PedsQL-CM scale followed a three-step linguistic validation method proposed by the Mapi Research Institute, a leading organization in linguistic validations of patient-reported assessments. In the forward translation stage, we invited two assistant professors who specialized in caring for children with CHD, both native Chinese speakers and bilingual in English with Ph.D. degrees from the US, to independently perform the forward translation. Then, the research team discussed the most appropriate translations of items, instructions, and response choices.

A Canadian professional translator who is bilingual in Chinese conducted a backward translation. The research team reviewed the content of the backward translation and returned with opinions and questions if there were any ambiguities. This iterative process was finished when all sentences of the backward translation were conceptually equivalent to the original version of the scale. The result of backward translation was sent to the original author, and the research team revised the traditional Chinese version according to the comments from the original author until the conceptual equity was achieved. In the patient-testing stage, face-to-face cognitive interviewing and the ‘think aloud’ methods were used in five pairs of adolescent patients and their parents to examine the question answering process of the respondents to find and refine the descriptions that were not identified during the translation process. Interviews were audio-recorded and transcribed verbatim. All sentences were understood correctly.

#### PedsQL™ 4.0 generic core (PedsQL-GC) scale

The PedsQL-GC scale was used to examine the criterion-related validity of the PedsQL-CM scale [36]. The traditional Chinese version of PedsQL-GC has been translated and proved to be reliable and valid [37]. This scale encompasses 15 items with four factors: physical functioning (5 items), emotional functioning (4 items), social functioning (3 items), and school functioning (3 items). Each item is rated on a 5-point Likert scale. In this scale, items were reverse-scored and linearly transformed to a 0–100 scale. The physical health summary score was identical to the physical functioning subscale, while the mean psychosocial health summary score was calculated by dividing the summation of the items by the number of items in the emotional, social, and school functioning subscales. A higher average score revealed a higher level of HRQoL.

#### Statistical analysis

### Missing data imputation

Up to 0.0%. to 1.2% of missing data was found on the item level. Little’s test of missing completely at random (MCAR) indicated that the assumption of MCAR may hold (*p*-value = 0.276) [38]. All missing data were imputed by hot deck imputation, which avoids the normality assumption.

### Reliability and item analysis

Reliability was assessed by Cronbach’s alpha and McDonald’s omega for the total scale and each subscale. Cronbach’s alpha if one item was deleted and item-total correlation were also examined. A Cronbach’s alpha and McDonald’s omega ≥ 0.70 and an item-total correlation coefficient ≥ 0.30 suggested an acceptable level of internal consistency and reliability for the scale [39]. The analyses of self-reports and parent proxy-reports were conducted independently.

### Criterion-related validity

Following the approach in the original version, we examined the criterion-related validity through the intercorrelations between the PedsQL-CM and PedsQL-GC [16]. Heart problem-related items were compared with those related to physical functioning. Items of the physical appearance subscale were compared with those of psychosocial functioning. Cognitive problems and treatment anxiety items were compared with the subscales of school functioning and psychosocial functioning, respectively. A medium (0.30. to 0.49) and large (> 0.50) effect size indicated acceptable and good criterion-related validity, respectively [16].

### Construct validity

We conducted a second-order confirmatory factor analysis (CFA) to examine the factorial structure. The first-order factors were heart problems and treatment, perceived physical appearance, treatment anxiety, cognitive problems, and communication; the second-order factor was the HRQoL. We used the maximum likelihood estimation with Satorra-Bentler’s robust correction, which is recommended in the situation of nonnormality and ordinal scaling [40, 41], and has been suggested when the response categories were equal to or greater than five [42, 43]. A simulation study found that this robust estimation generally produced less biased standard error estimates and good recovery of the population interfactor correlations [44]. We evaluated the model fit with the absolute fit index Satorra-Bentler’s scaled ${\chi }^{2}$ and ${\chi }^{2}/df$. A nonsignificant ${\chi }^{2}$ test and ${\chi }^{2}/df$ less than three were used as criteria for the model fit. We further considered incremental indices such as comparative fit index (CFI), Tucker–Lewis index (TLI), and parsimony indices—root mean square error of approximation (RMSEA) and Standardized Root Mean Square Residual (SRMR). A cut-off score of 0.90 or higher for the CFI and TLI or < 0.10 for the RMSEA and SRMR showed an adequate model fit between the measurement model and the observed data [45, 46]. We checked the assumption of the local independence by examining the inter-item residual correlations. A value of residual correlation larger than 0.3 implied that some items might still be mutually correlated even if conditional on the latent factors [47].

### Measurement invariance

A second-order multi-group factor model in CFA was analyzed to validate the measurement invariance between adolescents with CHD and their parents. The parameters were estimated by the maximum likelihood estimation with Satorra-Bentler’s robust correction. We sequentially examined configural (M1), first-order metric (M2), first- and second-order metric (M3), first-order scalar (M4), and first- and second-order scalar invariance (M5) [34]. In addition to ${\chi }^{2}$ statistic, we considered other model fit indices for nested models with CFI, RMSEA, and SRMR. A change of less than 0.01 of ΔCFI and less than 0.015 of ΔRMSEA for nested models supported metric invariance (M1 vs M2 and M2 vs M3) and scalar invariance (M3 vs M4 and M4 vs M5). A criterion of the change of ΔSRMR less than 0.03 and 0.015 was used to test metric invariance and scalar invariance, respectively [35].

### Agreement between self-reports and parent proxy-reports

Agreement between self-reports and parent proxy-reports was analyzed at the group level and the individual level. Paired t-tests were used for group comparison of the observed scores between subscales and the total scale. Cohen’s *d* revealed a small effect size of more than 0.20, a medium effect size of 0.50–0.80, and a large effect size of $\ge$ 0.80 [48]. Values of Cohen’s *d* smaller than 0.20 indicated a negligible mean difference between adolescents and parents. The individual level of adolescent-parent agreement was analyzed by intraclass correlations (ICC), with the values of $\le$ 0.40 considered poor to fair, 0.40–0.75 considered moderate, and $\ge$ 0.75 considered excellent, respectively [49]. We further illustrate the adolescent-parent agreement with Bland–Altman plots [50] by demonstrating the relationship between the means of two reports and the difference between two reports. We added the lines of the 95% limit of agreement of the mean difference and the 95% confidence intervals of the upper and lower limits.

Data analysis was mainly performed using SAS version 9.4 for Windows, except that the ICC was estimated by SPSS version 27 for Windows and McDonald’s omega was calculated by MBESS package in R software version 4.2.0. A two-tailed *p*-value less than 0.05 was considered statistically significant.

## Results

### Sample characteristics

A total of 162 adolescents and 162 parents participated in this study (Table 1). The majority of the adolescents with CHD were female (55.6%), in junior high school (56.1%), had simple complexity CHD (52.5%), and had a diagnosis of atrial septal defect type II (ASD type II, 25.9%). Most of the participating parents were mothers (79.6%) with a median age of 45 years, and many had college or university education (45.1%).

**Table 1:** Sample characteristics of self-reports and parent proxy-reports

	Self-report (*n* = 162)	Proxy-report (*n* = 162)
Female	90 (55.6)	129 (79.6)
Median age	15	45
	(Q1 = 13; Q3 = 16)	(Q1 = 42; Q3 = 48)
Education level		
Elementary school	5 (3.1)	5 (3.1)
Junior high school	91 (56.1)	11 (6.8)
Senior high school	63 (38.9)	55 (34.0)
College/university	3 (1.9)	73 (45.0)
Graduate school	0 (0)	18 (11.1)
Complexity^a^		
Simple	85 (52.5)	
Moderate	43 (26.5)	
Complex	34 (21.0)	
Diagnosis		
Atrial septal defect (ASD), type II	42 (25.9)	
Tetralogy of Fallot (TOF)	20 (12.3)	
Ventricular septal defect (VSD)	16 (9.9)	
Pulmonary valve abnormality	15 (9.3)	
Transportation of the great arteries (TGA)	14 (8.6)	
Pulmonary atresia (PA)	11 (6.8)	
Others	44 (27.2)	

^a^The disease severity for adolescents with CHD was classified as simple, moderate, and complex [49]. Examples of simple severity were ventricular septal defects (VSDs), atrial septal defects (ASDs). Moderate severity mainly included transportation of the great arteries (TGA), tetralogy of Fallot (TOF), and pulmonary valve abnormality. Examples of complex CHD were pulmonary atresia, mitral atresia, and Eisenmenger syndrome

### Reliability and item analysis

As shown in Table 2, the values of Cronbach’s alpha and McDonald’s omega were larger than 0.70 in self and parent proxy-reports. The parent proxy-report had a higher level of internal consistency than self-report regardless of the total scale or subscales (0.82 to 0.95 vs. 0.71 to 0.88). The alpha values remained similar if one item was deleted. Furthermore, all of the item-total correlations were higher than 0.30.

**Table 2:** Internal consistency and item analysis of traditional Chinese PedsQL-CM

	Cronbach’s alpha		McDonald’s omega		Alpha value if one item deleted		Item-total correlation	
	Self-report	Proxy-report	Self-report	Proxy-report	Self-report	Proxy-reports	Self-report	Proxy-report
Heart problems and treatment	0.80	0.87	0.82	0.87	0.75- 0.81	0.84–0.87	0.36- 0.72	0.48–0.76
Perceived physical appearance	0.71	0.83	0.77	0.86	0.45–0.72	0.69–0.85	0.45–0.66	0.61–0.76
Treatment anxiety	0.85	0.95	0.86	0.95	0.77–0.88	0.91–0.98	0.67–0.81	0.76–0.94
Cognitive problems	0.82	0.89	0.82	0.90	0.76–0.80	0.85–0.90	0.55–0.69	0.62–0.83
Communication	0.82	0.82	0.82	0.83	0.65–0.84	0.67–0.85	0.59–0.78	0.61–0.77
Total scale	0.88	0.91	0.87	0.91	0.87–0.88	0.91–0.91	0.34–0.60	0.43–0.63

### Validity

**Fig. 1:**
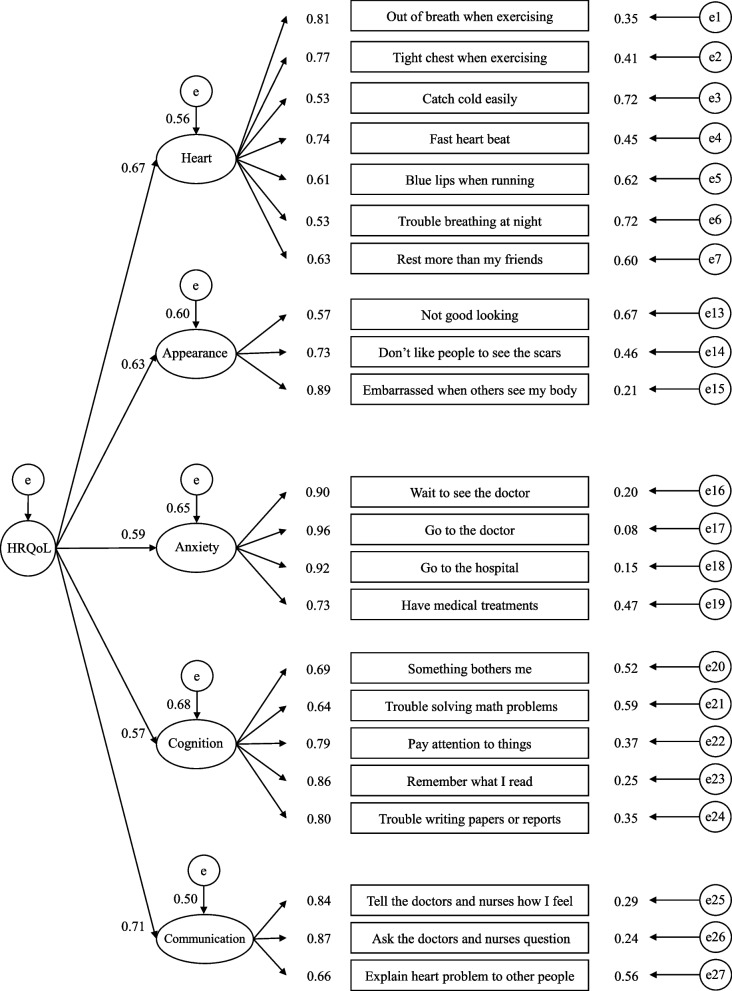
The factorial structure of traditional Chinese PedsQL-CM with standardized factor loadings

Both intercorrelations and the second-order factor model suggested the validity was acceptable. The hypothesized intercorrelations between the two scales were significant in medium to large effect size and in the expected direction, ranging from 0.34 to 0.77 for adolescents and from 0.46 to 0.68 for parents (Table 3). The second-order factor model demonstrated acceptable model fit to the data: Satorra-Bentler scaled ${\chi }^{2}=$ 290.9 (*p* < 0.001), *df* = 204, ${\chi }^{2}/df$ = 1.4, CFI = 0.967, TLI = 0.963, RMSEA = 0.036 (90% CI = 0.026–0.046), SRMR = 0.065. The inter-item residual correlations ranged from -0.14 to 0.22, indicating no violation of local independence. All standardized factor loadings were significant (Fig. 1).

**Table 3:** Intercorrelations between traditional Chinese PedsQL-CM and PedsQL-GC

	PedsQL-CM									
PedsQL-GC	Heart Problems		Perceived appearance		Treatment anxiety		Cognitive problems		Communication	
	Self-report	Proxy-report	Self-report	Proxy-report	Self-report	Proxy-report	Self-report	Proxy-report	Self-report	Proxy-report
Physical functioning	0.77***	0.66***	0.32***	0.31***	0.38***	0.30***	0.36***	0.38***	0.41***	0.35***
Psychosocial functioning	0.62***	0.60***	0.53***	0.46***	0.34***	0.48***	0.47***	0.66***	0.42***	0.52***
School functioning	0.54***	0.52***	0.31***	0.25**	0.26***	0.30***	0.46***	0.68***	0.28***	0.38***

Effect sizes are considered small (< .30), medium (.30—.49), or large (> .50)

^**^ *p* < 0.01

^***^ *p* < 0.001

## Measurement invariance

The result of measurement invariance between self-reports and parent proxy-reports is shown in Table 4. Initially, the estimated parameters in the configural model (M1) were allowed to vary freely between the two groups. The fit indices indicated that the configural model fitted the observed data well, showing that the self-reports and parent proxy-reports shared the same patterns of constructs. The scale achieved metric invariance based on ΔCFI, ΔRMSEA, and ΔSRMR (M1 vs M2 and M2 vs M3), indicating that each item contributed to the construct in a similar manner across groups. The M3, M4, and M5 were established to examine the first-order only and both first- and second-order scalar invariance. The results of ΔCFI, ΔRMSEA, and ΔSRMR (M3 vs M4 and M4 vs M5) supported the scalar invariance, showing that the magnitude of each item intercept was to a similar degree across groups. Therefore, measurement invariance across adolescents and parents was confirmed in the observed data.

**Table 4:** Measurement invariance across self-reports and parent proxy-reports of the traditional Chinese PedsQL-CM

Model	${\upchi }^{2}$	*df*	CFI	RMSEA	90% CI	SRMR	Comparison (Δ)	ΔCFI	ΔRMSEA	ΔSRMR
M1	485.6***	408	0.972	0.034	(0.020, 0.046)	0.073	–	–	–	–
M2	505.6***	425	0.971	0.034	(0.020, 0.045)	0.079	M1 vs M2	0.001	0.000	0.006
M3	511.5***	429	0.970	0.035	(0.021, 0.046)	0.086	M2 vs M3	0.001	0.001	0.007
M4	544.9***	446	0.964	0.037	(0.025, 0.048)	0.087	M3 vs M4	0.006	0.002	0.001
M5	554.9***	450	0.962	0.038	(0.026, 0.048)	0.089	M4 vs M5	0.002	0.001	0.002

M1: Configural invariance

M2: Metric invariance of the first-order factors

M3: Metric invariance of the first- and second-order factors

M4: Scalar invariance of the first-order factors

M5: Scalar invariance of the first- and second-order factors

^***^: The chi-square test was significant with *p* < 0.001

The cut-off criteria were ≤ .10 for ΔCFI, ≤ .15 for ΔRMSEA, ≤ .03 for ΔSRMR testing metric invariance, and ≤ .015 for ΔSRMR testing scalar invariance

## Adolescent-parent agreement

**Fig. 2:**
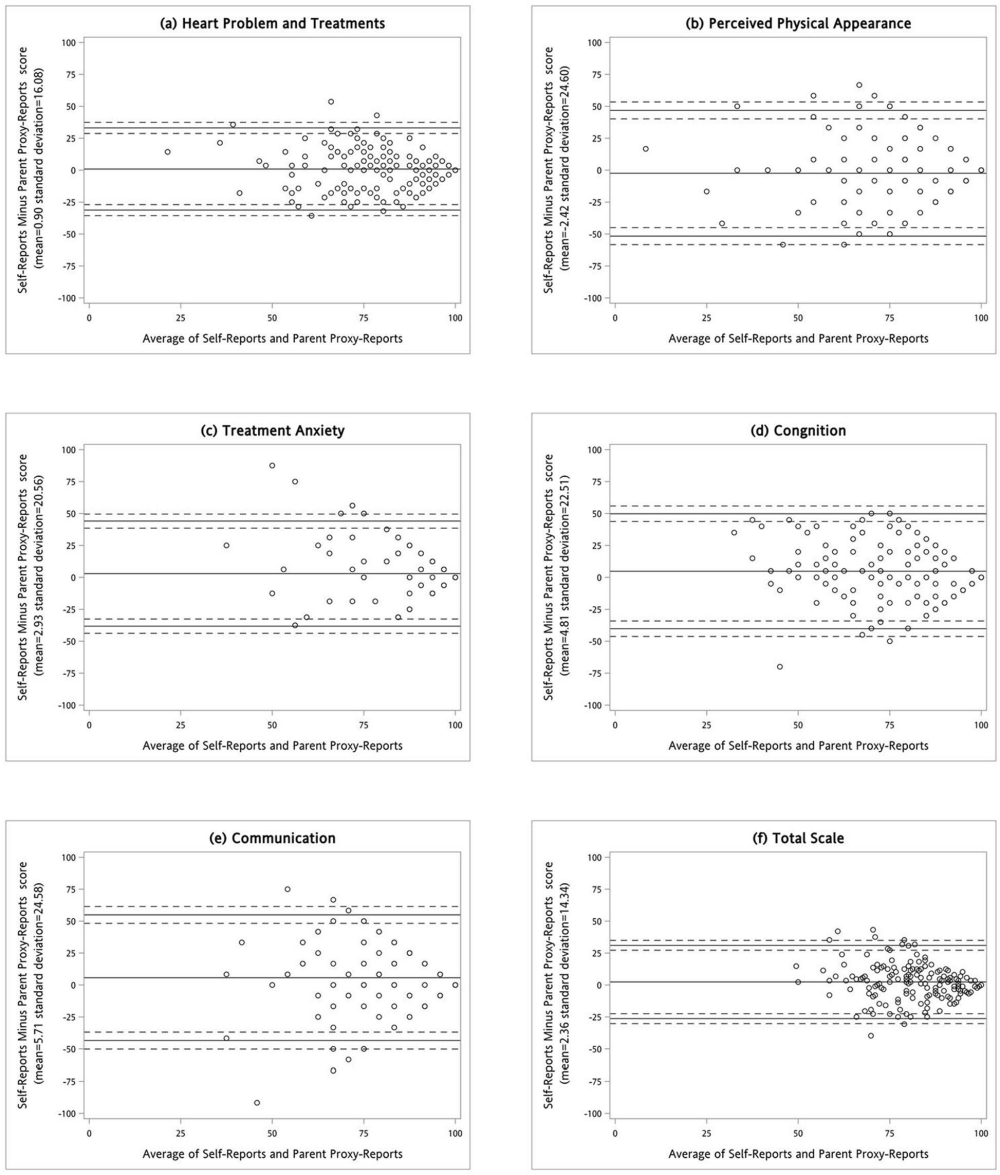
Bland–Altman plots for self-reports and parent proxy-reports of traditional Chinese PedsQL-CM (The solid lines represent the mean of difference and its 95% of limit of agreement. The dash lines represent the 95% confidence intervals of the upper and lower limit of agreement)

The analyses of the adolescent-parent agreement are shown in Table 5. At the group level, there was a negligible difference in total HRQoL between self-reports and parent proxy-reports (*p*-value = 0.038, Cohen’s *d* = 0.16). The adolescents with CHD rated a significantly higher level of HRQoL in cognitive problems and communication subscales than their parents. Although the *p*-value of treatment anxiety was borderline (*p*-value = 0.071), its effect size was negligible (Cohen’*d* = 0.14). At the individual level, the largest adolescent-parent ICC was for the heart problems and treatment subscale (ICC = 0.70), whereas the lowest ICC was for the communication subscale (ICC = 0.27). All Bland–Altman plots presented the rightwards arrow shapes (Fig. 2). Most adolescent-parent dyads fell into the 95% limits of agreement, with narrower ranges in the heart problem and treatment subscale and the total scale.

**Table 5:** Agreement of traditional Chinese PedsQL-CM between self-reports and parent-proxy reports

Subscale	Self-report (Mean $\pm$ SD)	Proxy-report (Mean $\pm$ SD)	Mean difference	Cohen's *d*	*p*-value^§^	ICC
Heart problems and treatment	79.0 $\pm$ 15.9	78.1 $\pm$ 17.3	0.9	0.06	0.475	0.70***
Perceived physical appearance	80.3 $\pm$ 21.3	82.8 $\pm$ 22.1	-2.4	0.10	0.213	0.53***
Treatment anxiety	90.8 $\pm$ 14.3	87.9 $\pm$ 18.5	2.9	0.14	0.071	0.37**
Cognitive problems	79.3 $\pm$ 18.0	74.5 $\pm$ 21.8	4.8	0.21	0.007**	0.52***
Communication	86.1 $\pm$ 18.4	80.3 $\pm$ 19.5	5.7	0.23	0.004**	0.27*
Total scale	82.4 $\pm$ 12.0	80.0 $\pm$ 14.1	2.4	0.16	0.038*	0.57***

*SD* Standard deviation

^§^ *p*-value for the paired t-test

^*^ *p* < 0.05

^**^ *p* < 0.01

^***^ *p* < 0.001

## Discussion

A series of PedsQL™ scales have been developed to accommodate disease-specific pediatric populations and are used worldwide. We evaluated the psychometric properties of the traditional Chinese version of the PedsQL-CM except for the drug-related treatment subscale. We found that PedsQL-CM has acceptable reliability and validity to assess disease-specific HRQoL in adolescents with CHD aged 12–18. Scalar invariance and adolescent-parent agreement in the total scale were noted, implying that parents could be surrogates for rating the total HRQoL for adolescents.

Although the adolescent version of PedsQL-CM was initially developed for 13 to 18 years, we also recruited 14 twelve-year-old adolescents. When those adolescents were recruited, some were junior high students in the first semester of the first year. The others had just graduated from elementary school and were waiting to enter junior high school. We included those adolescents in the statistical analyses since early adolescence approximately corresponds to junior high school years [51]. We performed a subgroup analysis on 13 to 18 years, and the conclusions remained unchanged.

The level of internal consistency of the CHD adolescents and their parent responses was satisfactory for group comparison. Parent proxy-reports had better internal consistency than self-reports, which was consistent with previous reports using the Swedish, Hungarian, Italian, and Brazilian translated versions [17, 19–21]. In terms of the item analysis, although one study indicated that the item-total correlation coefficients were low in the heart problem and treatment subscale [20], we found that no item needed to be deleted from the scale. Therefore, it can be inferred that the PedsQL-CM has adequate feasibility and reliability.

For criterion-related validity, we found that the highest correlated subscale with the treatment anxiety domain in self-reports was the physical functioning instead of the expected psychosocial functioning, and, interestingly, the correlation between the treatment anxiety domain and the physical functioning was the lowest in the Brazilian version [20]. The cognitive problems subscale was also found to have almost an equally high correlation with psychosocial functioning rather than school functioning, which was also found in the Hungarian version [17]. However, these comparisons were not discussed in the original version [16]. The incongruent findings might result from the study population. Our study focused on the adolescent population, while the others recruited participants aged 2–18 years [17, 20]. In addition, only our study population is Asian among the original and translated versions. Although we speculated that the factorial structures might differ to some extent across cultures, to our knowledge, no other studies examined the factorial structure or the measurement invariance across cultures for PedsQL-CM. Therefore, this speculation can only be validated in future studies. Furthermore, these findings demonstrated that PedsQL-CM might not be perfectly correlated with PedsQL-GC. However, a low level of intercorrelation may not guarantee that PedsQL-CM is invalid. Future studies may use additional scales to examine the criterion-related validity of PedsQL-CM.

It is possible that some parents mistakenly answered as standard parent reports instead of parent proxy-reports no matter if researchers had introduced how to complete the scale. It may be one possible cause why adolescent-parent disagreements were found in some studies since the constructs are methodologically different. In fact, parent proxy-reports had a higher correlation and closer average scores with adolescent self-reports compared with standard parent reports [52]. To avoid mistaken conclusions, we examined measurement invariance across self-reports and parent proxy-reports to ensure that the observed differences were not due to the discrepancy in factorial structures. Overall, the consistent evidence from three different analyses for adolescent-parent agreement implied the feasibility of the parent proxy-report for assessing the total HRQoL in clinical practice, which was consistent with the results in the traditional Chinese version of PedsQL-GC [53]. Healthcare providers can provide both instruments to assess the generic and cardiac-specific HRQoL. Although the observed difference in total HRQoL score was significant, the effect size was poor, indicating that the total difference between adolescents and parents was negligible. A similar phenomenon has been reported by Ooi et al. [29], where the child-mother total HRQoL difference in the obese population was significant but with no minimal clinically important difference. Therefore, statistical significance should not be the only criterion for child-parent agreement.

Special attention is required when interpreting the specific subscales from parent proxy-reports. We found that parents were more likely to rate lower scores for their adolescents in cognitive problems and communication subscales. Although studies have not shown consistent findings, some demonstrated a similar direction of discrepancy in cognitive problems and communication domains as revealed in this study [19, 32]. Adolescents and parents showed a higher agreement in observable domains and lower agreement in non-observable domains. This tendency was consistent with previous studies [17, 32]. The Bland–Altman plots with a rightward arrow shape showed greater variability in non-observable domains, which can also be found in PedsQL-GC [54]. Interestingly, although the disagreement in cognition problems subscale was noted in the group comparison, its magnitude of ICC was moderate (ICC = 0.52). The inconsistent findings using different analyses were likewise noticed in a previous study [55]. Therefore, the adolescent-parent agreement should be examined using multiple statistical analyses to make a robust conclusion. Future studies can implement other statistical models to investigate the related factors for the adolescent-parent discrepancy, such as actor–partner interdependence models.

As advocated in previous studies, parent proxy-reports sometimes provide additional information helpful for knowing the HRQoL of adolescents [56, 57]. From a developmental perspective, adolescents may sometimes be unwilling to discuss their thoughts on sensitive topics. In such scenarios, parent proxy-reports are appropriate to measure the perceived HRQoL of adolescents. Culturally, adolescents in Asian countries have heavy homework in school, and many go to cram schools in the evenings or on weekends. Parents are usually the deputy persons for regular visits, especially in those adolescents who have mild CHD. In general, healthcare providers could prioritize self-reports, but we suggest providing both self-reports and parent proxy-reports if allowed. Not only could parent proxy-report reveal adolescents’ HRQoL to some extent, but it could also offer healthcare providers an opportunity to understand the thoughts of their parents and adjust the treatment plan if needed.

Some limitations need to be noted in our study. First, we only focused on adolescents with CHD in this study, so the results cannot be directly extended to other age groups or pediatric cardiac disease. Second, we used a cross-sectional study design and did not evaluate test–retest reliability and longitudinal invariance. Thus, longitudinal studies are recommended in the future to obtain additional psychometric properties. Third, we did not analyze HRQoL about drug-related treatment in this study as only the adolescents who were currently taking medication needed to complete the subscale. At last, most parent proxy-reports were completed by mothers, and the adolescent-parent agreement may differ when fathers are proxies [29]. However, mothers were often reported as the primary care providers [58] and have been reported to be the majority of the proxies [59, 60], indicating that our results are representative of the real-world situation.

## Conclusion

The traditional Chinese version of PedsQL-CM shows acceptable reliability and validity for assessing the disease-specific HRQoL in adolescents with CHD. Adolescents and their parents interpret this scale in a conceptually similar manner. Parents may be proxies for adolescents with CHD to rate total HRQoL. When the patient-reported score is the primary outcome, the proxy-reported score could serve as a secondary outcome for research and clinical evaluation.

## Data Availability

Not applicable.

## Abbreviations

ASD: Atrial septal defect
CFA: Confirmatory factor analysis
CFI: Comparative fit index
CHD: Congenital heart disease
CI: Confidence interval
HRQoL: Health-related quality of life
ICC: Intraclass correlation
MCAR: Missing completely at random
PA: Pulmonary atresia
PedsQL-CM: Pediatric Quality of Life™ 3.0 Cardiac Module
PedsQL-GC: Pediatric Quality of Life™ 4.0 Generic Core
RMSEA: Root mean square error of approximation
SD: Standard deviation
SRMR: Standardized Root Mean Square Residual
TGA: Transportation of the great arteries
TLI: Tucker–Lewis index
TOF: Tetralogy of Fallot
VSD: Ventricular septal defect
